# Current Status of Air Toxics Management and Its Strategies for Controlling Emissions in Taiwan

**DOI:** 10.3390/toxics4020008

**Published:** 2016-04-12

**Authors:** Wen-Tien Tsai

**Affiliations:** Graduate Institute of Bioresources, National Pingtung University of Science and Technology, Pingtung 912, Taiwan; wttsai@mail.npust.edu.tw; Tel.: +886-8-770-3202; Fax: +886-8-774-0134

**Keywords:** air toxics, air quality management, regulatory system, human carcinogen, dioxins, heavy metal

## Abstract

Since the 1970s, hazardous air pollutants (HAPs), so-called air toxics, have been of great concern because they can cause serious human health effects and have adverse effects on the environment. More noticeably, some of them are known to be human carcinogens. The objective of this paper is to investigate the regulatory systems and human health effects of air toxics which have been designated by the Taiwan government under the Air Pollution Control Act. These toxic air pollutants include acutely toxic gas (*i.e*., ammonia, chlorine, fluorides, hydrochloric acid, hydrogen cyanide, hydrogen sulfide, nitric acid, phosphoric acid and sulfuric acid), gas containing heavy metals, and carcinogenic chemicals (including formaldehyde, vinyl chloride, asbestos and matter containing asbestos, dioxins and furans, volatile organic compounds, polycyclic aromatic hydrocarbons, and polychlorinated biphenyls). In line with international concern about the carcinogenic risk and environmental persistence of polychlorinated dibenzo-*p*-dioxins and polychlorinated dibenzofurans (PCDDs/PCDFs) and heavy metals in recent years, the current status in monitoring and reducing the emissions of PCDDs/PCDFs from stationary sources was analyzed as a case study in the present study. Furthermore, the control strategies for reducing emissions of air toxics from stationary sources in Taiwan were also addressed.

## 1. Introduction

Criteria air pollutants and toxic air pollutants represent two kinds of hazardous substances present in the atmosphere. Hazardous air pollutants (HAPs) or so-called air toxics have been recognized as one kind of principal air pollutant in the atmosphere since the 1970s. These toxic air pollutants could be associated with adverse human health effects [1,2], including cancer, reproductive effects, and respiratory illness. For example, the high incidences of leukemia, liver cancer and lung cancer have occurred in the urban environment [3,4], showing a large number of carcinogenic air pollutants such as benzene, vinyl chloride, and benzo(a)pyrene (one of polycyclic aromatic hydrocarbons). As a consequence, there is an additional control program, which focuses on the human health effects from exposure to air toxics other than the criteria air pollutants in the ambient environment. For example, a group of carcinogenic chemicals were initially specified as HAPs in the National Emission Standards for Hazardous Air Pollutants (NESHAP) under the U.S. Clean Air Act Amendments of 1970 and 1977, including asbestos and beryllium in 1973, vinyl chloride in 1975, benzene in 1977, and inorganic arsenic in 1980 [5].

According to the definition by the U.S. Environmental Protection Agency (US EPA), the term HAPs may be used interchangeably with air toxics, which may be reasonably anticipated to pose a threat of adverse effects on human health including cancer, reproductive effects, birth defects, and respiratory illness [5]. In this regard, air toxics should include a variety of toxic air pollutants that are reflected in the modes of action when exposed to them, causing acute illness such as nausea and other respiratory injuries, and chronic diseases such as carcinogenicity, tumorigenicity and teratogenicity [2]. These designated air toxics could be released from major sources, which include petrochemical facilities, motor vehicles, metal-processing industries, fossil-fuel-fired power plants, and waste incineration plants [5]. As a result, the ambient air levels (AALs) or ambient air guidelines for specific air toxics have been developed as part of the overall air quality management [6]. For example, the Ministry of the Environment of Japan set the environmental quality standards of priority air toxics (*i.e.*, benzene, trichloroethylene, perchloroethylene, dichloromethane and dioxins), and also promotes countermeasures (voluntary measures) by business operators. 

In recent years, it was found that air toxics (especially in dioxins) emissions in Taiwan are higher than those of other countries [7,8]. The central competent authority, the Environmental Protection Administration of Taiwan (Taiwan EPA), have been devoted to controlling the emissions of dioxins and heavy metals since the mid-1990s. These toxic substances present in the atmosphere may be grouped into persistent organic pollutants (POPs) and endocrine-disrupting chemicals (EDCs). More significantly, there was no literature addressing the air toxics management in Taiwan. This comprehensive paper will focus on the air toxics management in Taiwan, in line with international concern about the human health risk. This article is a brief overview to give the following key elements:

- Regulatory systems of controlling air toxics in Taiwan
- Carcinogenic and non-carcinogenic health risks of designated air toxics
- Current status: emissions of heavy metals and dioxins from stationary sources
- Control strategies for the emissions of air toxics from stationary sources in Taiwan

## 2. Regulatory Systems of Controlling Air Toxics in Taiwan

In Taiwan, the basic law governing and promoting air pollution control and prevention is the Air Pollution Control Act (APCA), which was initially passed in May 1975 and recently amended in May 2006. The goal of this act is to prevent and control air pollution, safeguard public health, protect against air quality deterioration, and improve the living environment. The APCA indicates the types of air pollutants including gaseous pollutants, particulate pollutants, secondary pollutants, toxic pollutants (air toxics) odor pollutants, and other substances designated by the central competent authority (*i.e*., the Taiwan EPA). Under the definition of the Act, toxic pollutants can be divided into carcinogenic and non-carcinogenic air pollutants, which are specified as follows: fluorides, chlorine gas (Cl_2_), ammonia gas (NH_3_), hydrogen sulfide (H_2_S), formaldehyde (HCHO), gas containing heavy metals, gaseous sulfuric acid/nitric acid/phosphoric acid/hydrochloric acid, vinyl chloride monomer (VCM), polychlorinated biphenyls (PCBs), hydrogen cyanide (HCN), dioxins and furans, carcinogenic polycyclic aromatic hydrocarbons, carcinogenic volatile organic compounds (VOCs), and asbestos and matter containing asbestos. Under the authorization of the Act, there are some regulations concerning the emission standards of air pollutants (including air toxics) from stationary sources based on specially designated industry categories, facilities, pollutant types or zones. The stationary sources include waste incineration, lead secondary smelting, sintering (steel industry), steel-making and casting furnace, spray-drying (ceramics industry), glass-making, open tunnel kiln (brick and tile industry), asphalt-mixing, electricity generation, hot air drying, surface finishing (automotive industry), cement, semiconductor, polyurethane (PU) synthetic leather , and dry cleaning.

It should be noted that VOCs not only have high potential for photochemical ozone creation, but also can cause acute and chronic health effects, such as carcinogenicity. As a consequence, the Taiwan EPA also promulgated the Air Pollution Control and Emission Standards for Volatile Organic Compounds. On the other hand, PCDDs/PCDFs have been listed in the POPs under the Stockholm Convention due to their adverse effects on human health and the environment [9]. During the past two decades (1994–2014), there have been 24 large-scale municipal solid waste (MSW) incineration facilities operated in Taiwan, implying that PCDD/PCDF emissions from these incinerators are serious. Therefore, the Taiwan EPA initially promulgated the PCDD/PCDF emission standard for MSW incinerators in August 1997 under the authorization of the APCA. Basically, the emission standards stipulate that the dioxin level of incinerator stack must comply with the standard limit of 0.1 ng TEQ/m^3^, which may be the most stringent regulation on PCDD/PCDF control in the world [10].

## 3. Carcinogenic and Non-Carcinogenic Health Risks of Designated Air Toxics

### 3.1. Carcinogenic Health Risks of Designated Air Toxics

Based on epidemiological reports [11], high cancer risks from air toxics exposure have been strongly supported. These toxic air pollutants, such as benzene, have been detected in the ambient air at low concentrations, and it is a concern that long-term exposure to such pollution would cause negative effects on human health, especially in cancer and tumor formation. As a result, the Air Pollution Control Act specially defines air toxics in the Enforcement Rules. More importantly, many of these designated air toxics are carcinogenic to humans. By compiling data from the international agencies such as the International Agency for Research on Cancer (IARC), the National Toxicology Program (NTP) of the U.S. Department of Health and Human Services, and the American Conference of Governmental Industrial Hygienists (ACGIH) [12,13,14], Table 1 lists the confirmed human carcinogens found in air toxics designated in Taiwan. Herein, IARC, NTP and ACGIH categorize the confirmed human carcinogens as “Group 1—carcinogenic to humans”, “known to be a human carcinogen” and “A1—confirmed human carcinogen”, respectively. It is obvious that these carcinogenic PCDDs/PCDFs, PAHs and VOCs should include benzene, benzo(a)pyrene (one of the PAHs), 1,3-butadiene, chloromethyl methyl ether (*i.e*., bis(chloromethyl)ether), 1,2-dichloroproane, formaldehyde, 2,3,4,7,8-Pentachlorodibenzofuran (one of the PCDFs), 2,3,7,8-tetrachlorodibenzo-*p*-dioxin (one of the PCDDs), and trichloroethylene, according to the classification of confirmed human carcinogens.

### 3.2. Non-Carcinogenic Health Risks of Designated Air Toxics

Based on the previous description, the non-carcinogenic air toxics include NH_3_, Cl_2_, fluorides, HCl, HCN, H_2_S, HNO_3_, H_3_PO_4_ and H_2_SO_4_ because of their acute effects on human health. Table 2 summarizes the acute (non-carcinogenic) health hazards according to the threshold limit value (TLV) basis developed by the ACGIH [14]. These toxic air pollutants are similar in health hazards because of their chemical properties such as corrosiveness (highly acidic) and the reactivity in the physiological effects. These gaseous chemicals irritate the respiratory system and skin and/or eyes. Thus, the inhalation of relatively low concentrations of these gases and vapors will cause an unpleasantness, and a pungent sensation, followed by a feeling of suffocation, cough, and a sensation of constriction in the chest. It was also noted that some of the designated air toxics (*i.e*., NH_3_, HNO_3_, H_3_PO_4_ and H_2_SO_4_) in Table 2 are not listed in the category of HAPs compiled by the US EPA. The data in Table 2 illustrate that inhalation is the most likely way to be exposed to air toxics. Notably, fluorides may be present in the forms of hydrogen fluoride, fluoride ion, and hydrofluoric acid. As a consequence, fluorosis or chronic fluorine intoxication has been correlated with fluoride deposition in the skeletal tissues (*i.e*., teeth and bone) of both animals and humans on the basis of clinical and epidemic studies [15].

## 4. Current Status: Emissions of Heavy Metals and Dioxins from Stationary Sources

In the face of the Stockholm Convention on POPs, the Taiwanese government has been devoted to establishing interactive models for the environmental surveillance and monitoring and destruction and reduction technologies in recent years. Based on the EPA survey by the Taiwan Air Quality Monitoring Network (website: http://taqm.epa.gov.tw/taqm/en/), the total emissions of dioxins from the stationary sources area were estimated to be 327.4 g I-TEQ in 2002, and they gradually decreased to 52.6 g I-TEQ in 2013, indicating a reduction rate as high as 84%. It should be noted that there are five types of air quality monitoring stations (*i.e*., general, industrial, traffic, national park, and background) in the Network. As shown in Table 3 [17], the average, minimum and maximum concentrations of ambient air monitoring of dioxins in 2013 were 0.031, 0.002 and 0.149 pg I-TEQ/m^3^, which was significantly reduced as compared to 0.089, 0.03 and 0.207 pg I-TEQ/m^3^ in 2002, respectively. All the concentrations at industrial and traffic air quality monitoring stations, even the highest measured value during the period of 2002–2013 (*i.e*., 0.238 pg TEQ/m^3^), were much lower than Japan’s "Environment Quality Standards for Dioxins" 0.6 pg TEQ/m^3^ (annual average), which was announced on December 27 1999. The air quality standard may be the only reference value in the world. Herein, dioxins include PCDDs, PCDFs and coplanar polychlorinated biphenyls (PCBs). The decreasing trend of dioxins emissions can be closely correlated to the promulgation of the regulatory systems. According to the inventory of dioxins in Taiwan [17], the main emission sources include waste incinerators, electric arc furnaces of the steel-making industry, sinter plants of the steel-making industry and the ashes thermal refining facilities for the steel-making industry. Therefore, the government adopted the most advanced pollution control equipment (e.g., dioxins capture by activated carbon adsorption, high-efficiency baghouse filters) in the incinerators to meet the most stringent emission standards for dioxins. Also, the Taiwan EPA has promulgated several relevant regulations for air pollution control and emission standards of dioxins from the above-mentioned stationary facilities since 1997. 

Regarding the ambient air monitoring concentrations of toxic metals in recent years, the average concentrations of arsenic, cadmium and nickel were 1.93, 0.48 and 4.41 ng/m^3^, respectively, which are well below the European Commission’s air quality standards (*i.e*., 6, 5 and 20 ng/m^3^, respectively, based on the annual average). These target values entered into force on December 12 2012. In order to control the release of heavy metals into the atmosphere, there are several relevant regulations concerning the air pollution emission standards of lead (Pb)/cadmium (Cd)/mercury (Hg) and their compounds from stationary sources, including lead secondary smelting facilities and waste incineration plants. To be in compliance with the international regulatory trends, the Taiwan EPA revised "the Air Pollutant Emissions Standards of Waste Incineration Plants" on December 25 2006, indicating that the emission standards for lead (0.2 mg/Nm^3^), cadmium (0.04 mg/Nm^3^), mercury (0.05 mg/Nm^3^) and other heavy metals were rigorous, with an over 10-fold increase in comparison with the original emission standards (lead for 10 mg/Nm^3^, cadmium for 1 mg/Nm^3^) in 1992.

Over the past 10 years, the emission standards of dioxins and heavy metals in Taiwan have been tightened by Taiwan EPA and local governments. As described above, the emissions of air toxics have been effectively reduced under the inter-ministerial cooperation and on-site audits. In addition to process control and operating conditions for the maintenance of air pollution control equipment, the proper source reduction can also mitigate air toxics emissions. For example, scrap metal sorting management (e.g., cable-containing polyvinyl chloride excluded) can avoid potential dioxin precursor substances from high-temperature processing systems, which decreased emissions, then lessening the environmental and health risks. 

## 5. Control Strategies for the Emissions of Air Toxics from Stationary Sources in Taiwan

### 5.1. Control Measures

The central competent authority (*i.e*., Taiwan EPA) will continuously develop, conduct or revise the industry-specific emission standards under the authorization of the APCA, and also promulgate the following measures:

- Expand the public and private facilities as mandatory premises, which are requested to apply for emission permits to install, modify, or operate their stationary sources.
- Require newly established stationary pollution sources to adopt better measures for reducing the emissions of particulate matters (*i.e*., PM_2.5_) and VOCs with the use of the best available control technology (BACT) approaches.
- Reinforce the control of dioxins (including PCBs, PCDDs and PCDFs) and other air toxics (especially heavy metals) that are listed as POPs.
- Require all gas stations to be equipped with gasoline vapor recovery systems because various types of toxic volatile organic compounds (e.g., benzene) have been detected in the ambient air, and there are concerns about the photochemical smog formation and the health hazards of exposure to carcinogenic air pollutants.
- Monitor the fugitive emission levels of highly reactive VOCs and air toxics from petrochemical industries and refineries using open-path Fourier transform infrared spectroscopy, and also upgrade or improve the performance of their existing air pollution control devices.
- Subsidize local governments to perform air quality improvement programs (e.g., clean air zone, green vehicles promotion) under the funding of the air pollution control fee. The Taiwan EPA also organized the Air Pollution Control Advisory Taskforce to provide guidance and evaluate the status of implementation.

### 5.2. Establishing a Sound Air Pollution Control Fee System

In recent years, ozone (O_3_) has become the most important air pollutant based on the monitoring results of the pollutant standards index (PSI) in Taiwan. It is well known that the ozone formation is mainly derived from the photochemical reactions of VOCs and nitrogen oxides (NOx) under strong solar radiation. As a result, the Taiwan EPA revised the fee rates; that is, VOCs are added into the fee system and stationary sources with massive emissions of NOx and VOCs are requested to pay reasonable fees according to different air quality control zones, specific VOCs types and emission status in comparison with emission standards. For example, the emissions of some toxic VOCs, including benzene, ethylbenzene, styrene, methylene chloride, 1,1-dichloroethane, 1,2-dichloroethane, chloroform, 1,1,1-trichloroethane, carbon tetrachloride, trichloroethylene and perchloroethylene (*i.e*., tetrachloroethylene), will be requested to pay an additional fee rate at about US $1.0/kg.

On the other hand, based on the polluter pays principle, the Taiwan EPA will enforce the incentive measures to provide subsidies or deductions from air pollution control fees for industrial facilities that install or renew pollution prevention and control equipment. For example, pump station pumps, seals, valves, storage tanks, compressors, and flanges are main sources of fugitive VOC emissions in the petrochemical factories and refineries. The most efficient measures to reduce these emissions involve process improvements (*i.e*., process changes, equipment changes) to prevent their discharge, or collecting fugitive vapors from processing systems in local exhaust ventilation hoods for subsequent treatment or abatement.

## 6. Conclusions and Recommendations

The information about the air toxics management in Taiwan has been extensively described and analyzed in this paper. It focused on the regulatory systems and carcinogenic/non-carcinogenic health risks of air toxics, and the control strategies for their emissions from stationary sources. Due to the large number of large-scale MSWs and industrial furnace facilities (e.g., steel-making and petrochemical industries), the Taiwan government began adopting prevention policies and regulatory measures to enhance and review the control mechanisms for lessening the environmental and health risks posed by heavy metals and dioxins in recent years. It showed that the emissions of PCDDs/PCDFs in Taiwan are significantly decreasing.

Based on the case study of the risk management of air toxics in Taiwan, the following recommendations are further addressed:

1. Although there are 14 types of air toxics designated in Article 2 of APCA, their specific compounds regarding gases containing heavy metals, carcinogenic PAHs and VOCs should be specially defined by the Taiwan EPA.
2. The Taiwan EPA should set the air quality guidelines or standards for carcinogenic air toxics as policy objectives to satisfy these levels. The target air toxics could include organic compounds (e.g., benzene, formaldehyde, vinyl chloride and trichloroethylene) and compounds containing heavy metals (e.g., cadmium, chromium VI, nickel).
3. In compliance with the APCA, the Taiwan EPA and local governments should monitor carcinogenic air toxics in the atmosphere at regular intervals. In order to satisfy the right to know, these monitoring results should be compiled and open to the public.
4. To avoid the extensive use in market, the inter-ministerial efforts should establish green mark standards for these toxic-containing (e.g., vinyl chloride and formaldehyde) products, especially daily commodities, household plastics and apparel textures.
5. Biomonitoring and environmental monitoring are scientific methods for assessing human exposures to carcinogenic air toxics and potential health risks based on the sampling and analysis of human body fluids. When these measuring data are used, the population and possible exposure routes can be used to analyze their risk assessments for human beings, which would be further confirmed to reduce or prevent future exposure possibilities.

## Figures and Tables

**Table 1:** The classification of human carcinogens for the air toxics designated in Taiwan.

Air toxics	HAPs by US EPA	Confirmed human carcinogen		
		IARC	USNTP	ACGIH
Asbestos and matter containing asbestos	Listed	v	v	v
Benzene	Listed	v	v	v
Benzo(a)pyrene ^a^	Listed	v	-	-
Beryllium and beryllium compounds	Listed	v	v	v
1,3-Butadiene	Listed	v	v	-
Cadmium and cadmium compounds	Listed	v	v	-
Chloromethyl methyl ether	Listed	v	v	v
Chromium VI compounds	Listed	v	v	v
Formaldehyde	Listed	v	v	-
Nickel compounds	Listed	v	v	v
2,3,4,7,8-Pentachlorodibenzofuran ^b^	Listed	v	-	-
Polychlorinated biphenyls	Listed	v	-	-
2,3,7,8-tetrachlorodibenzo-*p*-dioxin ^c^	Listed	v	v	-
Trichloroethylene	Listed	v	-	-
Vinyl chloride	Listed	v	v	v

^a^ One of polycyclic aromatic hydrocarbons (PAHs); ^b^ One of polychlorinated dibenzo-*p*-furans (PCDFs); ^c^ One of polychlorinated dibenzo-p-dioxins (PCDDs).

**Table 2:** The non-carcinogenic human impairments of the air toxics designated in Taiwan using the ACGIH-TLV basis.

Air toxics	HAPs by US EPA	TLV basis by ACGIH ^a^
Ammonia (NH_3_)	-	Eye damage; URT irritation
Chlorine (Cl_2_)	Listed	URT & eye irritation
Fluorides (as F)	-	Bone damage; fluorosis
Hydrochloric acid (HCl)	Listed	URT irritation
Hydrogen cyanide (as cyanide compounds)	Listed	URT irritation; headache; nausea; thyroid effects
Hydrogen fluoride (HF)	Listed	(URT, LRT, skin & eye irritation; fluorosis) ^b^
Hydrogen sulfide (H_2_S)	Listed	URT, LRT, skin & eye irritation; headache; nausea; apnea
Nitric acid (HNO_3_)	-	URT & eye irritation; dental erosion
Phosphoric acid (H_3_PO_4_)	-	URT, eye & skin irritation
Sulfuric acid (H_2_SO_4_)	-	Pulmonary function

^a^ URT: upper respiratory tract; LRT: lower respiratory tract; ^b^ The health hazard information in parentheses was obtained from the NIOSH website [16].

**Table 3:** Environmental monitoring levels for dioxins during the years of 2002–2013 in Taiwan ^a^.

Dioxins	Environmental Monitoring Levels ^b^								
	2002	2006	2007	2008	2009	2010	2011	2012	2013
Average	0.089	0.034	0.047	0.049	0.048	0.051	0.039	0.029	0.031
Minimum	0.030	0.001	0.003	0.004	0.005	0.005	0.007	0.003	0.002
Maximum	0.207	0.133	0.183	0.210	0.135	0.238	0.232	0.132	0.149

^a^ Source [17]; ^b^ Unit: pg I-TEQ/m^3^.
